# Systematic Confirmation Study of GWAS-Identified Genetic Variants for Kawasaki Disease in A Chinese Population

**DOI:** 10.1038/srep08194

**Published:** 2015-02-03

**Authors:** Jiao Lou, Rong Zhong, Na Shen, Xu-zai Lu, Jun-tao Ke, Jia-yu Duan, Yan-qi Qi, Yu-jia Wang, Qing Zhang, Wei Wang, Fang-qi Gong, Xiao-ping Miao

**Affiliations:** 1 Department of Epidemiology and Biostatistics and Key Laboratory of Environment and Health, Ministry of Education & Ministry of Environmental Protection, and State Key Laboratory of Environmental Health (Incubating), School of Public Health, Tongji Medical College, Huazhong University of Science and Technology, Wuhan, PR China; 2 Guangdong Women and Children Hospital, Guangzhou, PR China; 3 Children's Hospital, Zhejiang University School of Medicine, Hangzhou, PR China; 4 Centre Hospitalier de l'Université de Montréal, CRCHUM-Hôpital Notre-Dame, Pavillion DeSève, Montreal, Canada

## Abstract

Genome-wide association studies (GWASs) have identified multiple single nucleotide polymorphisms (SNPs) associated with Kawasaki disease (KD). In this study, we replicated the associations of 10 GWAS-identified SNPs with KD in a Han Chinese population. Odds ratios (ORs) and 95% confidence intervals (CIs) were calculated by logistic regression, and cumulative effect of non-risk genotypes were also performed. Although none of the SNPs reached the corrected significance level, 4 SNPs showed nominal associations with KD risk. Compared with their respective wild type counterparts, rs1801274 AG+GG genotypes and rs3818298 TC+CC genotypes were nominally associated with the reduced risk of KD (OR = 0.77, 95% CI = 0.59–0.99, *P* = 0.045; OR = 0.74, 95% CI = 0.56–0.98, *P* = 0.038). Meanwhile, rs1801274 GG genotype, rs2736340 CC genotype or rs4813003 TT genotype showed a reduced risk trend (OR = 0.57, 95% CI = 0.35–0.93, *P* = 0.024; OR = 0.46, 95% CI = 0.26–0.83, *P* = 0.010; OR = 0.64, 95% CI = 0.43–0.94, *P* = 0.022), compared with rs1801274 AG+AA genotypes, rs2736340 CT+TT genotypes or rs4813003 TC+CC genotypes, respectively. Furthermore, a cumulative effect was observed with the ORs being gradually decreased with the increasing accumulative number of non-risk genotypes (*P*_trend_<0.001). In conclusion, our study suggests that 4 GWAS-identified SNPs, rs2736340, rs4813003, rs3818298 and rs1801274, were nominally associated with KD risk in a Han Chinese population individually and jointly.

Kawasaki disease (KD; OMIM 611775), also called Mucocutaneous Lymph Node Syndrome, is an acute febrile illness that preferentially affects children younger than 5 years old^1^. The major clinical manifestations of KD include prolonged fever, bilateral non-purulent conjunctivitis, diffuse mucosal inflammation, polymorphous skin rashes, peripheral extremity changes and cervical lymphadenopathy^2^,^3^. Pathologically, KD is a vasculitis of small and medium-sized arteries, and the coronary arteries are predominantly affected^4^. Coronary artery lesions, such as dilatation and aneurysm, develop in 15–25% of untreated and 3–5% of treated children^5^,^6^, making KD the leading cause of acquired childhood heart disease in developed countries.

KD occurs worldwide and is more common in East Asian populations, such as Japanese^7^, Koreans^8^ and Taiwanese^9^, with the incidence of 239.6, 113.1 and 66.2 per 100,000 children younger than five years old respectively, based on the latest nationwide survey. In China, the annual incidence rate of KD is at a range of 7.1–49.4 per 100,000 children younger than 5 years old, according to the recent epidemiologic studies conducted in several provinces^10^. Furthermore, the incidence rate and the total number of patients with KD have been continuously increasing.

Although the etiology of KD remains ambiguous, clinical and epidemiology evidences indicate that a ubiquitous infectious factor triggers an inflammatory response, resulting in host immune dysregulation in a small subset of genetically predisposed children^11^. For decades, great effort has been paid on seeking potential genes conferring KD, and the advent of genome-wide association studies (GWASs) has revolutionized the identification of genomic regions associated with the disease. Until now, a total of 6 published GWASs conducted in different ethnicities have identified multiple novel candidates for KD susceptibility. Burgner et al.^12^ performed the first GWAS of KD in a Dutch Caucasian population and subsequent fine-mapping stage confirmed 8 susceptibility genes, among which, 4 genes (*LNX1*, *CSMD1*, *ZFHX3*, *CAMK2D*) were involved in a plausible biological network and 5 genes (*LNX1*, *CSMD1*, *CAMK2D*, *NAALADL2*, *TCP1*) had decreased transcript abundance in the acute phase of illness. A GWAS conducted by Kim et al.^13^ in Korean and Taiwanese populations revealed 1p31 (rs527409) as one susceptibility locus for KD. A total of 10 SNPs located in 3 novel loci (*COPB2*, *ERAP1*, *IGHV*) were found to be associated with KD in a Han Chinese population residing in Taiwan, which was the first KD GWAS conducted in this population^14^. Another GWAS performed in Europeans and Asians identified that 2 loci (*FCGR2A*, *MIA-RAB4B*) contributed to KD risk^15^. Coincidentally, there were 2 GWASs of KD published online in the same journal and at the same time, one of which was conducted in a Han Chinese population residing in Taiwan, and reported 2 new susceptibility loci (*BLK*, *CD40*)^16^; the other one identified 3 new risk loci (*FAM167A-BLK*, *HLA*, *CD40*) in Japanese subjects^17^.

Considering the diversity of genetic architecture among ethnicities, the findings from other races could not truly represent the genetic susceptibility of KD in Chinese. Moreover, the KD research on Chinese are very important because of the high prevalence, but so far only one combined analysis of these GWAS loci has been performed in a Han Chinese population in Southwest of mainland China^18^. Therefore, we carried out a replication study on the association between GWAS-identified SNPs, alone and in accumulation with KD risk in another Han Chinese population in Southeast of China.

## Results

### Characteristics of study participants

A total of 428 KD patients and 493 healthy controls were enrolled in this replication study. The male to female ratios of cases and controls were 1.59 (263: 165) and 1.59 (303: 190), respectively, and no statistically significant difference was observed between cases and controls in the distribution of gender (Pearson *χ*^2^ = 0.000, *P* = 0.997).

### Association analysis between individual SNP and KD risk

The call rates of all the 10 SNPs genotyped were >95%, and the genotypes for all SNPs in controls conformed to Hardy-Weinberg equilibrium (HWE, *P*>0.005) and were similar to those in the HapMap database (http://hapmap.ncbi.nlm.nih.gov/, HapMap Data Rel 24/phase II Nov08, on NCBI B36 assembly, dbSNP b126), Han Chinese in Beijing, China (CHB) population (Table 1). As shown in Table 2, codominant, dominant, recessive and additive models were all performed for every SNP. Unfortunately, all the *P* values did not surpass the Bonferroni threshold in the association tests. However, among the 10 investigated SNPs, 4 SNPs were not significantly but nominally associated with KD susceptibility. Compared with the respective wild type counterparts, *FCGR2A* rs1801274 AG+GG genotypes and *TCP1* rs3818298 TC+CC genotypes were nominally associated with the reduced risk of KD (Odds ratio (OR) = 0.77, 95% confidence interval (CI) = 0.59–0.99, *P* = 0.045; OR = 0.74, 95% CI = 0.56–0.98, *P* = 0.038), the former of which has been reported in our another article^19^. Meanwhile, individuals carrying the *FCGR2A* rs1801274 GG genotype, *BLK* rs2736340 CC genotype or *CD*40 rs4813003 TT genotype showed a reduced risk trend of KD (OR = 0.57, 95% CI = 0.35–0.93, *P* = 0.024; OR = 0.46, 95% CI = 0.26–0.83, *P* = 0.010; OR = 0.64, 95% CI = 0.43–0.94, *P* = 0.022), compared with rs1801274 A allele carriers (AG+AA genotypes), rs2736340 T allele carriers (CT+TT genotypes) or rs4813003 C allele carriers (TC+CC genotypes), respectively. Under additive model, 3 SNPs (*FCGR2A* rs1801274, *BLK* rs2736340 and *CD40* rs4813003) were nominally associated or marginally associated with the reduced KD risk (OR = 0.76, 95% CI = 0.62–0.94, *P* = 0.010; OR = 0.81, 95% CI = 0.65–1.00, *P* = 0.052; OR = 0.80, 95% CI = 0.66–0.97, *P* = 0.023). What's more, the trend of the nominal association was consistent with that in the previous study where the corresponding SNP was identified, except the SNP rs3818298. The different ethnic groups or ethnicity-linked haplotype structure between Caucasians and Han Chinese might be taken into account (minor allele frequency (MAF) in Han Chinese versus Caucasians: 0.411 versus 0.192, downloaded from the online database of HapMap).

For the 4 SNPs nominally associated with KD susceptibility, we calculated the power for our sample size to detect an OR of 1.50, with an estimated average incidence of KD of 28.25/100,000 in China^10^. As a result, the statistical power before (after) multiple comparisons (significant level α = 0.05; α' = 0.001) for rs1801274, rs3818298, rs2736340 and rs4813003 was 84.3% (37.3%), 86.4% (40.6%), 79.5% (30.6%) and 85.5% (39.0%), respectively.

### Cumulative effect of rs1801274, rs3818298, rs2736340 and rs4813003 on KD risk

Next, we examined the cumulative effect of the 4 nominally significant SNPs by counting the number of non-risk genotypes associated with KD risk in each subject according to the potential inheritance models presumed by the results of dominant and recessive models from individual SNP analysis. For example, for rs1801274 and rs3818298, the non-risk genotypes were GA/GG and CT/CC genotypes, respectively; for rs2736340 and rs4813003, the non-risk genotypes were CC and TT genotypes, respectively. Accordingly, the other genotypes of the 4 SNPs were considered as risk genotypes. As a result, there was a gradual decrease in KD risk with the increasing accumulative number of non-risk genotypes after adjustment for gender (*P*<0.001 for Cochran-Armitage trend test). Compared with individuals carrying none of non-risk genotypes (that was, four risk genotypes), the ones who carried 3~4 non-risk genotypes had a significant association with reduced risk of KD (OR = 0.27, 95% CI = 0.14–0.53, *P*<0.001, Table 3).

## Discussion

The advances of high-throughput genotyping technologies and the increases of consortiums or biobanks of either population cohorts or case-control samples have created a new era of molecular genetics, and have made it a reality to perform rapid and efficient genotyping for hundreds of thousands of genetic variants without knowing gene function through GWAS^20^. In the past few years, the GWAS strategy has made great contribution to the genetic research on KD. As far as we know, 6 GWASs with a dozen of susceptibility loci for KD have been published^12^,^13^,^14^,^15^,^16^,^17^. In the present study, we systematically evaluated 10 identified SNPs in a hospital-based, case-control study in a Chinese population. We found that 4 SNPs (*FCGR2A* rs1801274, *TCP1* rs3818298, *BLK* rs2736340 and *CD40* rs4813003) were not significantly but nominally associated with KD risk in our study population, and the trend of each association was also consistent with that in the previous study where the corresponding SNP was identified, except one SNP rs3818298, which might be on account of the ethnic difference. In addition, a cumulative effect of the 4 SNPs was observed.

The SNP rs2736340 with the lowest *P* value in our study is located in the linkage disequilibrium (LD) region of the promoter and the first intron of *BLK* gene at 8p23.1. *BLK* encodes B-lymphoid tyrosine kinase, a Scr family tyrosine kinase expressed primarily in the B cell lineage, and transduces signals downstream following stimulation of B cell receptors^21^. B-cell receptor signaling is important for establishing the B-cell repertoire during development of these cells^22^ and plays a critical role in B-cell activation and antibody secretion. Recently, a replication in populations of Korean and European descent and meta-analysis of *BLK* rs2736340 have validated that the risk T allele was associated with lower expression of *BLK* in peripheral blood B cells during the acute stage of KD, thus altering B cell function and predisposing individuals to KD^23^, which was in consistent with our results. Furthermore, the rs2736340 was also found as a newly identified rheumatoid arthritis risk SNP by a GWAS^24^. As it happens, the KD GWAS conducted by Onouchi et al.^17^ reported the same loci, at which the identified SNP rs2254546 was in high LD with rs2736340 (D' = 1 and *γ*^2^ = 0.949 in the HapMap Japanese in Tokyo (JPT) and CHB populations). Another SNP rs13277113, which has been repeatedly proved associated with autoimmune diseases, such as systemic lupus erythematosus^25^,^26^ and systemic sclerosis^27^, was also in high LD with rs2736340 (D' = 1 and *γ*^2^ = 0.957 in the HapMap JPT and CHB populations). All of the above provided compelling evidence that autoimmunity and antibody-mediated immune responses might be involved in pathogenesis of KD.

rs4813003, located 4.9 kb downstream of *CD40*, was also nominally associated with KD risk in our study, and the trend of the association conformed to the previous meta-analysis^18^. CD40 is a member of the tumor necrosis factor receptor superfamily, and is expressed on antigen-presenting cells, such as B cells, macrophages and dendritic cells, and on vascular endothelial cells. Together with its ligand, CD40L, which is expressed on activated CD4^+^ T-helper cells, CD40 plays a pivotal role in the activation of both humoral and cellular immunity^28^. A functional SNP within the Kozak sequence of the *CD40* gene (rs1883832) was previously reported to alter the translation efficiency of *CD40*^29^, and was associated with increased risk of Grave's disease^30^,^31^,^32^,^33^, rheumatoid arthritis^34^ and acute coronary syndrome^35^. The SNP we studied was in moderate LD with rs1883832 (D' = 1 and *γ*^2^ = 0.570 in the HapMap JPT and CHB populations), while rs1569723, another susceptibility SNP at *CD40* locus identified by one KD GWAS^16^, was in high LD with rs1883832 (D' = 1 and *γ*^2^ = 0.953 in the HapMap JPT and CHB populations). More importantly, it has been suggested that the expression of CD40L on CD4^+^ T cells and platelets correlated to the coronary artery lesions and disease progression in KD^36^. These findings support the plausibility of our observation of the association between *CD40* rs4813003 and KD risk, although the biological mechanism awaits further investigation.

This study is the first to test the association of *TCP1* rs3818298 with KD risk in an independent sample set since its first identification by Burgner et al.^12^. *TCP1*, located at 6q25.3, encodes a molecular chaperone that is a member of the chaperonin containing TCP1 complex, also known as the TCP1 ring complex^37^, which has been shown to interact with and structurally fold actin and tubulin^38^. Several studies have indicated that TCP1 might contribute to neuropathological abnormalities, such as Down syndrome^39^,^40^, Alzheimer's disease^41^ and schizophrenia^42^, however, little is known about the correlation between TCP1 and KD, which needs more research in the future. Another SNP replicated in our study, rs1801274, which is a functional polymorphism in *FCGR2A* gene, encodes the H131R substitution. More details about this SNP and KD risk has been discussed in our another article^19^.

In addition, we did not observe any association between the other 5 SNPs and risk of KD. Among which, the inconsistent results obtained from the present study and previous study conducted by Burgner et al.^12^ might contribute to the ethnic discrepancy in study populations with the considerable differences in the allele frequencies of these SNPs between Chinese and Caucasians, such as rs9937546, rs1870740 and rs4834340. With regards to the other 2 SNPs, rs2233152 and rs2857151, the latter of which has been validated in a meta-analysis^18^, the insufficient statistical power due to the insufficient sample size might be taken into account.

Our study has several strengths. Firstly, the study was performed in a Han Chinese population, an ideal population for the replication study due to its high prevalence of KD. Moreover, some of the SNPs studied in this manuscript have already been associated with KD in Han Chinese population. For example, the association between SNP rs1801274 in *FCGR2A* gene and KD risk in Han Chinese subjects (Hong Kong, Shanghai and Taiwan) was assessed and the same trend of association was reported in the replication phase of one GWAS paper^15^, and afterwards Yan et al.^18^ validated the association in the Southwest area of the China mainland. Besides, SNP rs28493229 in *ITPKC* gene identified in earlier study^43^ and in tight LD with rs2233152 has already been replicated in several studies including those from China^44^. As with rs1801274, rs2233152 itself has also been associated in Han Chinese subjects (Hong Kong, Shanghai and Taiwan). What's more, another SNP rs1569723 in *CD40* region has been identified associated with KD at genome-wide significance level in one GWAS paper conducted in Han Chinese population residing in Taiwan^16^. Secondly, 3 out of the 4 loci validated in our current work were involved in immune system, which was therefore in accordance with the current consensus regarding KD pathogenesis^11^. Thirdly, we assessed the cumulative effect of nominally risk SNPs, which might improve the understanding of the role of genetic variants in KD susceptibility.

Despite of the strengths mentioned above, several limitations in the present study should be taken into consideration. Firstly, not all SNPs identified by GWASs were included in our study, thus it might not be comprehensive to some extent. Secondly, the sample size of this study was not so large that the statistical power was limited, and no significant associations were found with the significance level corrected by Bonferroni method for multiple comparisons. Therefore, caution should be taken in interpreting the negative and nominal results. Finally, lacking information of environment factors, such as family history and infection history, which might play roles in KD onset, limited our further research on gene-environment interactions.

In conclusion, our study suggests that 4 of the 10 GWAS-identified SNPs are nominally associated with KD risk in a Chinese population individually and jointly. Even though the associations were not significant, such information might still be helpful for further research on KD etiology and pathogenesis. More replication studies with larger sample size and functional studies are needed in the future research.

## Methods

### Study participants

The case population consisted of 428 KD patients consecutively enrolled from Children's Hospital, Zhejiang University School of Medicine, China from April 2009 to September 2012. All the cases were unrelated Han Chinese children and the diagnosis of KD was based on the 5th revised edition of the guidelines established by the Kawasaki Disease Research Committee in Japan in 2002^45^. The controls contained 493 ethnic- and gender-matched healthy children with no evidence of infection at the time of a routine health examination from the same hospital with cases.

This study was approved by the ethics committees of the Children's Hospital, Zhejiang University School of Medicine, and the methods were carried out in accordance with the approved guidelines. Participants or their parents/caregivers provided their written informed consent to join in this research.

### SNP selection and genotyping

At the beginning, we set the inclusion criteria of candidate SNPs at genome-wide significance with combined *P* < 5.0 × 10^−8^, under which circumstances, 5 risk loci identified by GWAS were included^15^,^16^,^17^. Then we selected one SNP from each of the 5 KD susceptibility loci, including rs1801274 in *FCGR2A*, which has been validated by a case-control study and subsequent integrated meta-analysis in our another article^19^, rs2233152 in *MIA-RAB4B* region, rs2857151 in *HLA-DQB2-HLA-DOB* region, rs4813003 in *CD40* region and rs2736340 in *BLK* region. In addition, we included 6 genes (*NAALADL2*, *CAMK2D*, *CSMD1*, *LNX1*, *TCP1* and *ZFHX3*) from the study performed by Burgner et al., which was the first GWAS of KD. Among the 6 genes, 4 genes (*LNX1*, *CAMK2D*, *ZFHX3* and *CSMD1*) were found consisted in a single functional network, with functional relationships potentially related to inflammation, apoptosis, and cardiovascular pathology. Besides, 5 genes (*CAMK2D*, *CSMD1*, *LNX1*, *NAALADL2*, and *TCP1*) had significantly differential expression when comparing the pairwise blood transcript levels during acute and convalescent KD^12^. Similarly, we selected one SNP with the MAF in CHB of >0.05 and the most significant *P* value from each of the 6 candidate genes. Considering that there was only one SNP in *CSMD1* gene (rs2912272), and the MAF in CHB was only 0.02, we excluded this locus as a consequence. Ultimately, a total of 10 SNPs from 10 susceptibility loci identified by GWASs were included in our replication study. Details of the investigated loci were summarized in Table 1.

Genomic DNA was extracted from 2 mL peripheral blood sample collected from each participant at recruitment, applying the RelaxGene Blood System DP319-02 (Tiangen, Beijing, China). The concentration and the optical density of DNA were confirmed by NanoDrop 1000 spectrophotometer (Thermo Fisher Scientific, Waltham, Massachusetts, USA). SNPs of each sample were genotyped by the TaqMan SNP Genotyping Assay (Applied Biosystems, Foster City, CA, USA) with a 7900 HT Fast Real-Time PCR System (Applied Biosystems, Foster City, CA, USA) according to the manufacturer's instructions. Genotyping was performed without knowing case control status, and a 5% random sample of cases and controls was genotyped twice by different investigators; the reproducibility was 100%. Moreover, quality control was performed by eliminating SNPs with a genotyping call rate of <95% and those that deviated from the HWE in controls.

### Statistical Analysis

The HWE for genotypes in controls was assessed by goodness-of-fit *χ^2^* test. Pearson's *χ^2^* test or Fisher's exact test was adopted to examine the differences of the distribution of gender and genotypes between cases and controls, when appropriate. The association between the case-control status and each SNP, measured by the OR and its corresponding 95% CI, was assessed by unconditional multivariable logistic regression with adjustment for gender. In order to avoid the assumption of genetic models, codominant, dominant, recessive and additive models were all calculated. Then for every nominally significant SNP, we divided the three genotypes into two groups, risk genotype and non-risk genotype, according to the potential inheritance model presumed by the results of dominant and recessive models in the individual SNP association analysis. We tested the cumulative effect of nominally significant SNPs by counting the number of risk genotypes in each subject. All of the statistical analyses above were conducted by SPSS v13.0 (SPSS, Chicago, Illinois, USA). LD was performed using the Haploview v4.2 software^46^, by determining D' and *γ*^2^ values. The statistical power to detect the associations of the SNPs was calculated by Power v3.0.0^47^,^48^. The significant levels were corrected with Bonferroni method in multiple comparisons (α' = α/10 = 0.005 for HWE; α' = α/10*5 = 0.001 for association analyses and power calculations), and a *P* value lower than the significant level was considered statistically significant in the analyses.

## Author Contributions

Conceived and designed the study strategy: X.M., F.G., W.W. Designed the experiment: J.L. Recruited the participants and collected their information and blood samples: J.L., X.L. Conducted the literature review and selected candidate SNPs: J.L., R.Z. Performed the experiments: J.L., N.S., X.L. Analysis the data: J.L., J.K., J.D., Y.Q. Wrote the manuscript: J.L. Prepared the tables and references: Y.W., Q.Z. All authors reviewed the manuscript.

## Figures and Tables

**Table 1:** Information of the 10 SNPs identified by GWAS studies of KD

Gene	rs ID	Position	MAF	MAF in controls	MAF in CHB†	Call rate (%)	*P* for HWE (α' = 0.005)‡
ZFHX3	rs9937546	16:71561220	C	0.051	0.067	98.59	0.505
NAALADL2	rs1870740	3:176364140	C	0.313	0.293	97.18	0.959
TCP1	rs3818298	6:160126706	C	0.445	0.411	99.78	0.050
CAMK2D	rs4834340	4:114620118	A	0.082	0.111	99.46	0.172
LNX1	rs6554112	4:54113392	G	0.309	0.386	99.57	0.287
FCGR2A	rs1801274	1:159746369	G	0.336	0.333	99.35	0.486
MIA-RAB4B	rs2233152	19:45972856	A	0.082	0.078	99.13	0.426
HLA-DQB-HLA-DOB	rs2857151	6:32871492	A	0.291	0.273	97.50	0.818
CD40	rs4813003	20:44196691	T	0.402	0.322	97.72	0.755
BLK	rs2736340	8:11381382	C	0.259	0.256	98.05	0.065

Abbreviations: MAF, minor allele frequency; CHB, Han Chinese in Beijing, China; HWE, Hardy-Weinberg equilibrium.

^†^MAF was downloaded from the online database of HapMap for Han Chinese in Beijing, China.

^‡^The significant level was corrected with the formula of α' = α/10 = 0.005 according to the Bonferroni method.

**Table 2:** Association between individual SNP and KD risk

rs ID		HW/HT/HV frequency		HT vs HW	HV vs HW	Dominant model	Recessive model	Additive model
	Ref/Var	KD cases	Controls	OR† (95%CI) *P*‡	OR† (95%CI) *P*‡	OR† (95%CI) *P*‡	OR† (95%CI) *P*‡	OR† (95%CI) *P*‡
rs1801274	A/G	212/185/27	213/226/52	0.82 (0.63–1.08) 0.157	**0.52 (0.31**–**0.86) 0.011**	**0.77 (0.59**–**0.99) 0.045**	**0.57 (0.35**–**0.93) 0.024**	**0.76 (0.62**–**0.94) 0.010**
rs1870740	T/C	201/181/35	226/205/47	1.00 (0.78–1.31) 0.972	0.84 (0.52–1.35) 0.471	0.97 (0.74–1.26) 0.797	0.84 (0.53–1.33) 0.460	0.95 (0.77–1.16) 0.598
rs6554112	A/G	182/202/41	240/200/52	**1.33 (1.01**–**1.75) 0.041**	1.04 (0.66–1.64) 0.866	1.27 (0.98–1.65) 0.072	0.90 (0.59–1.39) 0.646	1.12 (0.92–1.37) 0.249
rs4834340	G/A	351/74/1	411/78/1	1.11 (0.78–1.57) 0.554	1.17 (0.07–18.85) 0.913	1.11 (0.79–1.57) 0.549	1.15 (0.07–18.50) 0.923	1.11 (0.79–1.56) 0.549
rs3818298	T/C	149/205/72	141/265/87	**0.73 (0.55**–**0.98) 0.037**	0.78 (0.53–1.15) 0.216	**0.74 (0.56**–**0.98) 0.038**	0.95 (0.67–1.34) 0.765	0.86 (0.71–1.04) 0.115
rs2857151	G/A	206/180/30	243/197/42	1.08 (0.82–1.42) 0.600	0.84 (0.51–1.39) 0.503	1.04 (0.80–1.35) 0.797	0.81 (0.50–1.33) 0.408	0.98 (0.80–1.21) 0.878
rs2736340	T/C	254/151/17	272/169/40	0.96 (0.72–1.26) 0.749	**0.45 (0.25**–**0.82) 0.009**	0.86 (0.66–1.12) 0.265	**0.46 (0.26**–**0.83) 0.010**	**0.81 (0.65**–**1.00) 0.052**
rs9937546	T/C	385/36/0	439/46/2	0.89 (0.57–1.41) 0.625	NA	0.86 (0.54–1.35) 0.499	NA	0.83 (0.53–1.28) 0.391
rs2233152	G/A	355/62/4	413/77/2	0.94 (0.65–1.35) 0.724	2.33 (0.42–12.81) 0.331	0.97 (0.68–1.39) 0.876	2.35 (0.43–12.92) 0.325	1.01 (0.72–1.41) 0.949
rs4813003	C/T	173/201/47	173/227/79	0.88 (0.67–1.17) 0.395	**0.59 (0.39**–**0.90) 0.015**	0.81 (0.62–1.06) 0.123	**0.64 (0.43**–**0.94) 0.022**	**0.80 (0.66**–**0.97) 0.023**

Abbreviations: Ref, Reference allele; Var, Variant allele; HW, wild type homozygote; HT, heterozygote; HV, variant homozygote; KD, Kawasaki disease; OR, odds ratio; CI, confidence interval; NA, not available.

^†^OR calculation was conducted under assumption that variant alleles were risk alleles.

^‡^All the *P* values were adjusted for gender. The significant level was corrected with the formula of α' = α/10*5 = 0.001 according to the Bonferroni method. The nominal significant results were in bold.

**Table 3:** Cumulative effect of the 4 nominally significant SNPs between KD patients and normal controls

No. of non-risk genotypes	KD cases (%)	Controls (%)	OR	95%CI	*P*†
0	61 (14.88)	49 (10.49)	Ref.	Ref.	
1	178 (43.41)	181 (38.76)	0.79	0.52–1.22	0.288
2	153 (37.32)	184 (39.40)	0.67	0.43–1.03	0.069
3~4	18 (4.39)	53 (11.35)	**0.27**	**0.14**–**0.53**	**<0.001**
Cochran-Armitage Trend Test					**<0.001**

Abbreviations: CI, confidence interval; KD, Kawasaki disease; OR, odds ratio.

The 4 nominally significant SNPs were rs1801274, rs3818298, rs2736340 and rs4813003, and the respective non-risk genotypes were GA/GG, CT/CC, CC and TT genotypes.

^†^All the *P* values were adjusted for gender. The positive results were in bold.
